# Transcriptional Analysis of *Coccidioides immitis* Mycelia and Spherules by RNA Sequencing

**DOI:** 10.3390/jof7050366

**Published:** 2021-05-07

**Authors:** Aaron F. Carlin, Sinem Beyhan, Jesús F. Peña, Jason E. Stajich, Suganya Viriyakosol, Joshua Fierer, Theo N. Kirkland

**Affiliations:** 1 Department of Medicine, Division of Infectious Disease, U.C. San Diego School of Medicine, La Jolla, CA 92093, USA; acarlin@health.ucsd.edu (A.F.C.); sviriyakosol@gmail.com (S.V.); jfierer@health.ucsd.edu (J.F.); 2 J. Craig Venter Institute, La Jolla, CA 92037, USA; sbeyhan@health.ucsd.edu; 3 Department of Microbiology and Plant Pathology, Institute for Integrative Genome Biology, University of California-Riverside, Riverside, CA 92521, USA; jpena016@ucr.edu (J.F.P.); jason.stajich@ucr.edu (J.E.S.); 4 Infectious Diseases Section, VA Healthcare San Diego, San Diego, CA 92161, USA; 5 Department of Pathology, U.C. San Diego School of Medicine, La Jolla, CA 92093, USA

**Keywords:** *Coccidioides immitis*, coccidioidomycosis, fungus, dimorphic fungus, mycelium, spherule, differentiation, RNA-Seq, differential gene expression

## Abstract

*Coccidioides immitis* and *C.* *posadasii* are dimorphic fungi that transform from mycelia with internal arthroconidia in the soil to a tissue form known as a spherule in mammals. This process can be recapitulated in vitro by increasing the temperature, CO_2_ and changing other culture conditions. In this study, we have analyzed changes in gene expression in mycelia and young and mature spherules. Genes that were highly upregulated in young spherules include a spherule surface protein and iron and copper membrane transporters. Genes that are unique to *Coccidioides* spp. are also overrepresented in this group, suggesting that they may be important for spherule differentiation. Enriched GO terms in young spherule upregulated genes include oxidation-reduction, response to stress and membrane proteins. Downregulated genes are enriched for transcription factors, especially helix–loop–helix and C2H2 type zinc finger domain-containing proteins, which is consistent with the dramatic change in transcriptional profile. Almost all genes that are upregulated in young spherules remain upregulated in mature spherules, but a small number of genes are differentially expressed in those two stages of spherule development. Mature spherules express more Hsp31 and amylase and less tyrosinase than young spherules. Some expression of transposons was detected and most of the differentially expressed transposons were upregulated in spherules.

## 1. Introduction

*Coccidioides immitis* and *C.* *posadasii* are primary pathogenic fungi that are endemic in the desert regions of the Western United States, Mexico, and Central and South America [1]. They cause pulmonary infections that range from asymptomatic infections to severe pneumonias and can disseminate beyond the lung [2,3]. The organisms grows as a mold in the soil and produce asexual spores, termed arthroconidia, within the mycelium. When the soil is disturbed, the mycelia can rupture and the arthroconidia are released. If inhaled by a susceptible host, the arthroconidium differentiates into a very different and unique form that is known as a spherule. In tissue, the spherule enlarges and can form many reproductive endospores. Mature spherules rupture and release endospores, which can then differentiate into the next generation of spherules. This form, the spherule, is the disease-associated form of the organism. If the disease is self-limited, the reproduction of spherules is limited, but if the disease is severe, then spherules continue to grow, rupture and give rise to new spherules, while eliciting inflammatory and immune responses. This process goes on in many mammalian hosts, including desert rodents, which may be important in the ecology of the fungus [4].

The morphological transition between mycelia and spherule forms of *C. immitis* is dependent on sensing the host environment, and this transition can be recapitulated in the laboratory by changing the temperature and other growth conditions. *C. immitis* grow in saprobic form at 22–30 °C; culturing at 37–42 °C with 5–20% CO_2_ in Converse media is used to convert arthroconidia to spherules [5,6]. Utilizing these conditions, whole genome-level transcriptional profiling studies of saprobic and parasitic forms have been performed [7]. That work reported that about 1300 genes are upregulated in mycelia, about 1900 genes are upregulated in spherules, and expression of known virulence genes is upregulated in the spherules of both *C. immitis* and *C. posadasii*, linking morphology to the virulence traits. For example, the spherule outer wall glycoprotein, which is the outermost layer on the spherule, is expressed only in spherules [8,9]. Another study compared mycelia to young spherules and mature spherules to mycelia and to each other using microarray technology [10]. In the current study, we analyze the RNA obtained from frozen samples of spherules and mycelia obtained from the previous microarray study by strand-specific RNA sequencing. Our results show that there are more than 1500 genes that are differentially regulated in spherules and mycelial phases of *C. immitis*. We have compared the up- and downregulated genes to previous studies and, where possible, analyzed the function of differentially expressed genes.

## 2. Materials and Methods

### 2.1. Culture Conditions 

*C. immitis* RS strain was grown as mycelia or spherules as previously described [10]. To grow the mycelia, 2 × 10^6^ arthroconidia/mL were incubated in 250 mL flat-bottom Erlenmeyer flasks (Corning, Corning, NY, USA) in 50 mL of GYE media. The flasks were cultured in a 30 °C incubator without shaking for 5 days. To grow the spherules, arthroconidia were washed 2 times in modified Converse media. The spores were inoculated at 4 × 10^6^ arthroconidia/mL into a 250 mL baffled Erlenmeyer flask containing 50 mL of modified Converse media. The flasks were set up and grown on a shaker at 160 rpm, in 14% CO_2_ at 42 °C. Four flasks were harvested 2 days after inoculation and the remaining four flasks after 8 days. Fresh Converse media was not added. The spherules did not rupture and release endospores within that time in this culture system.

### 2.2. RNA Extraction, Purification and Sequencing

The mycelia and spherule samples were stored in QIAzol (Qiagen) at −70 °C for seven years. Samples were added to 2 mL ZR BashingBead lysis tubes with 0.5 mm beads (Zymo Research, Irvine, CA, USA) and the tubes were arranged in a pre-cooled Tissuelyzer II adapter (Qiagen, Germantown, MD, USA) and disrupted by shaking at 50 Hz for 25 min. Total RNA was purified from mycelia and spherule samples (2 replicates/condition) using chloroform extraction and isopropanol precipitation and quantified using a Qubit 3.0 Fluorometer (Invitrogen, Carlsbad, CA, USA). For RNA-Seq, strand-specific, paired-end libraries were prepared from total RNA by ribosomal depletion using the Yeast Ribo-Zero rRNA Removal Kit (Illumina, San Diego, CA, USA) and then using the TruSeq Stranded total RNA-Seq kit (Illumina) according to manufacturer’s instructions. Then, 100 bases were sequenced from both ends using an Novaseq 6000 instrument according to the manufacturer’s instructions (Illumina, San Diego, CA, USA). A total of 10 million reads per sample were acquired.

### 2.3. Analysis of RNA-Seq Data

Duplicate sets of paired strand-specific reads were mapped to predicted mRNA sequences and quantified using Kallisto [11]. Read count tables from the Kallisto output were analyzed by DESeq2 in R. The results were filtered for a Benjamini–Hochberg adjusted *p* value < 0.05; upregulation and downregulation for the gene tables are defined as more than 2_log2_ or less than −2_log2_ spherules/mycelia fold change (FC). This cut off is chosen to minimize the false positive identification of differentially regulated genes. All FC in Tables are expressed as log_2_ values; FC changes in the text are expressed as arithmetic values.

### 2.4. Functional Analysis of Genes

All differentially expressed genes were evaluated using data from FungiDB version 51 (https://fungidb.org/fungidb/app, accessed on 4 January 2021) [12]. Orthology searches were done using the orthology tool at FungiDB. The FungiDB tool compares two sets of proteins by reciprocal BLASTP and computes the percent match length with a threshold for orthology, where a blast match e value is < 10^−5^ with a percent match length of ≥ 50%. Finally, the FungiDB tool obtains paralogs, orthologs and ortholog groups using OrthoMCL Pairs release 6.4 (https://orthomcl.org, accessed on 4 January 2021) [13]. *C. immitis* genes without a BLASTP match with an e value < 10^−8^ to all fungal species other than *Coccidioides* spp. and less than ten orthologs were considered genus-specific. Multiple alignment of transcription factors was done with Clustal Omega (https://www.ebi.ac.uk/Tools/msa/clustalo/, accessed on 4 January 2021) [14]. GO enrichment analysis was done using FungiDB (https://fungidb.org/fungidb/app, accessed on 4 January 2021) and FungiFun (https://elbe.hki-jena.de/fungifun/, accessed on 4 January 2021) [15]. GO term enrichment using the FungiFun tool was deemed significant if the Benjamini–Hochberg adjusted *p* value was < 0.05). GO enrichment analysis using the FungiDB tool was considered significant if the *p* value was < 0.05. Enrichment was carried determined using a Fisher’s Exact test with the background defined as all genes from the organism being queried. *p*-values corrected for multiple testing are provided using both the Benjamini – Hochberg false discovery rate method and the Bonferroni method. GO term enrichment are presented in Supplemental Table S2.

The FungiDB pathway tool was used to generate data in Supplemental Table S3. The pathway enrichment analysis of differentially expressed genes was conducted using the tool at FungiDB, which searches for annotation at MetaCyC (https://metacyc.org/, accessed on 4 January 2021) [16] and KEGG (https://www.genome.jp/kegg/, accessed on 4 January 2021) [17].

## 3. Results and Discussion

**Figure 1:**
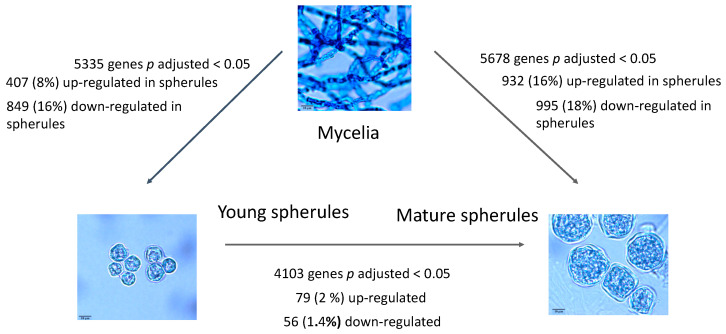
Differential expression of genes in mycelia, young and mature spherules. Legend Figure 1: mRNA was obtained from mycelia, young spherules and mature spherules for RNA-Seq. The morphology of the organism and the number of differentially expressed genes is shown.

The RNA-Seq gene expression from the three conditions tested were compared to each other and the results are shown in Figure 1 and Supplemental Table S1. Comparing young and mature spherules to mycelia, at least 8% of genes were upregulated and 16–18% were downregulated (FC > 2_log2_ or < −2_log2_, adjusted *p* value less than 0.05). The number of differentially expressed genes comparing young spherules to mature spherules was much smaller (1.4–2%).

### 3.1. Differential Gene Expression in Young Spherules vs. Mycelia

The 20 most differentially expressed genes in young spherules are shown in Table 1.

The most highly upregulated gene is the spherule outer wall glycoprotein (SOWgp). This protein is expressed in very large amounts on the external surface of spherules but not mycelia and is involved in pathogenicity [8,9]. Two other spherule antigenic proteins, expression-library immunization protein-1 and parasitic-phase-specific protein PSP-1, [18,19], are also very highly upregulated, as are three transporters, including a copper transporter. There are only three genes that are predicted to be copper transporters in *C. immitis*, so this is a substantial increase in transporter expression, which suggests that spherules may have a much higher need for copper than mycelia do. Recent experiments in *Paracoccidioides brasiliensis* showed that copper deprivation has a major effect on fungal metabolism, but similar experiments have not been done in *Coccidioides* spp. [20]. In addition, a comparison of gene expression in *C. posadasii* spherules and an engineered chitinase deletion that does not endosporulate found that a major difference was the upregulation of iron and copper uptake genes, which is further evidence for the importance of iron and copper uptake for spherule and endospore formation [21]. Three of the twenty very highly expressed genes are genus specific. Although this group of genes was highly upregulated, this does not imply that they are functionally related.

Examining all of the 408 upregulated genes in young spherules, there is no correlation between gene length and FC. Those that are genus specific are more common in differentially expressed genes than in the whole genome. A total of 24% of all *C. immitis* genes are genus specific compared to 36% of differentially expressed genes. This suggests that some genus-specific genes may be important for spherule differentiation (Table 2).

**Figure 2:**
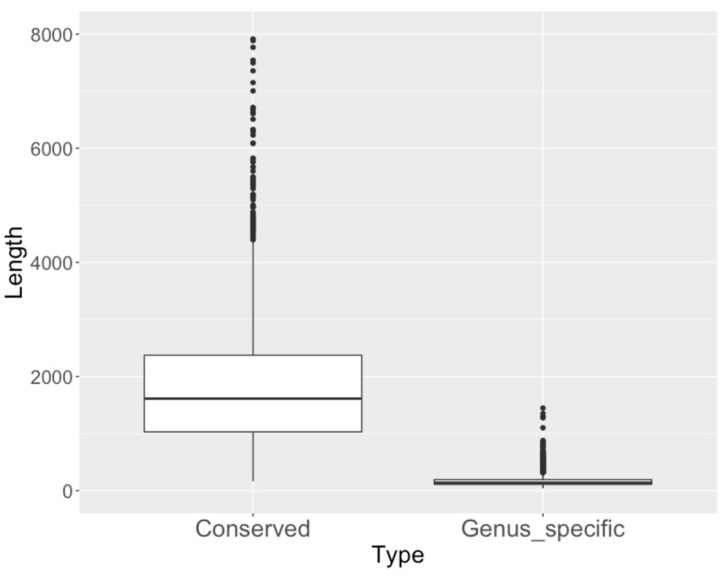
Length of genus-specific and conserved genes. Legend Figure 2: Lengths (*Y*-axis) of conserved and differentially expressed genus-specific proteins.

Out of all differentially expressed genus-specific genes, 11% have signal peptides (compared to 5% of all *C. immitis* genes), 7% have a transmembrane domain (compared to 16% of all genes) and 1.6% have a predicted Pfam domain (compared to 40% of all genes). Differentially expressed genus-specific genes were much shorter than conserved genes (Figure 2).

Enriched GO terms in the upregulated genes (young spherule/mycelia) included oxidation-reduction processes, integral membrane components, transmembrane transport and response to stress (Supplemental Table S2). One of the oxidation/reduction genes is superoxide dismutase, which has been shown to be important for pathogenicity in *Histoplasma capsulatum* [22]. Changes in expression of oxidation/reduction genes are to be expected since the atmosphere for mycelial culture is air with 0.4% CO_2_ compared to 14% CO_2_ in spherule culture conditions. Several metal transporters, including copper and iron transporters are also upregulated. A cluster of genes, including an iron siderophore, are induced by iron-deprivation and are required for pathogenicity in *H. capsulatum* [23]. Five of the six iron-related genes (including the siderophore biosynthesis cluster) have homologs in *C. immitis* that are upregulated in young spherules. These genes are tightly clustered (<25 kb) on contig 1 and have the upstream regulatory sites for the GATA transcription factor Sre1 that is identified in identified in *H. capsulatum*. The *Blastomyces dermatitidis* homolog of Sre1 has been knocked out and the resulting mutant is unable to differentiate from mold to yeast [24]. However, the *C. immitis* homolog of Sre1, SreP, is downregulated in young spherules. Taken together, these results show that the organization of iron-related genes in *C. immitis* and *H. capsulatum* is very similar and that most of the *C. immitis* iron acquisition genes are upregulated in both of these dimorphic fungi. Although this may imply the upregulation of spherules in vivo, in this experiment, mycelia and spherules were grown in different media, which may have played a role in the upregulation of iron-regulated genes in this experiment. 

Cellular component GO terms associated with cell membranes are dramatically overrepresented in upregulated genes (young spherule/mycelia) (*p* < 10^−4^). In addition, 22% of the up-regulated genes have at least one predicted transmembrane domain, compared to 17% of total genes (*p* < 0.05). However, there is no difference in the proportion of up-regulated genes compared to all genes with a predicted signal peptide.

A pathway enrichment analysis was also conducted (Supplemental Table S3). An analysis of upregulated genes (young spherule/mycelia) found that 24 pathways were enriched with a *p* value of less 0.01 but none had a Benjamini–Hochberg adjusted *p* value of less than 0.05. The most highly enriched pathways in upregulated genes were pentose and glucuronate interconversion (see Supplemental Table S3). Other enriched pathways included the degradation of proponate, furfural and benzoate. Two pathways for polyketide synthase were also enriched.

Many genes that have previously been identified to be upregulated in spherules were also found to be upregulated in this study. In addition to SOWgp [8,9] and the parasitic phase specific gene [19], expression of the urease and the ureidoglycolate hydrolase genes was also upregulated [25,26]. One gene that was found to be upregulated in the yeast (or spherule) phase of all dimorphic fungi is 4-hydroxyphenylpyruvate dioxygenase (4-HPPD or HpdA) [27]. This gene is involved in tyrosine catabolism, which plays a role in the synthesis of melanin [28]. Chemical inhibition of 4-HPPD blocks the formation of yeast in *P. brasiliensis* and deletion of the gene blocks’ differentiation to yeast [29]. There are two genes coding for 4-HPPD in *C. immitis*—one is upregulated and the other is downregulated. Boyce described a cluster of genes involved in tyrosine catabolism in *Pennicillium marneffei* and other pathogenic dimorphic fungi. The expression of this cluster of genes is upregulated when tyrosine is the only nitrogen source [28]. However, *C. immitis* spherules are grown in media containing ammonium salts as the primary nitrogen source.

There are twice as many downregulated genes in young spherules than are up-regulated, so many genes that are expressed well in mycelia are expressed poorly in spherules. Downregulated genes are enriched for the cytochrome p450 superfamily, transcription factors and genes with oxidoreductase GO terms (Table 3). There are two homologs of the cytochrome p450 ERG11 gene in *C. immitis*: CIMG_00573 and CIMG_07469. ERG11 is involved in azole anti-fungal activity in other fungi, and both of these are downregulated in young spherules. 

**Figure 3:**
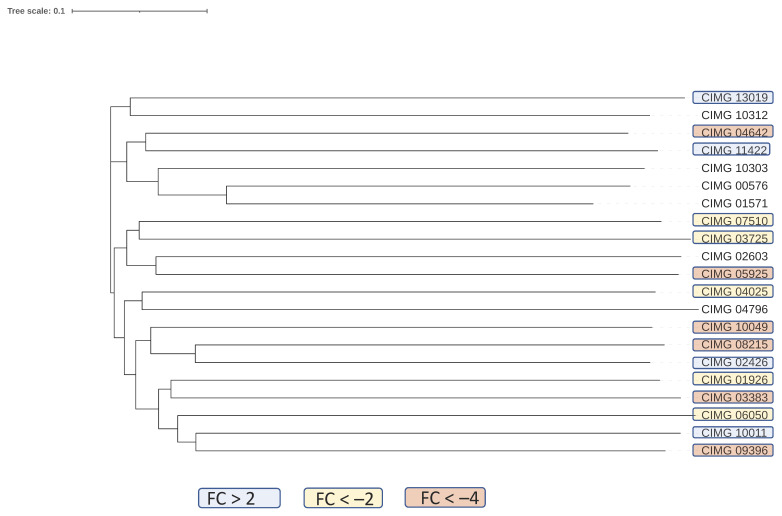
Phylogenetic tree of C2H2 transcription factors and FC. Legend: Phylogenetic tree was built on protein sequence. FC (young spherule/mycelium) values of *C. immitis* C2H2 transcription factors. Gene IDs within tan boxes are downregulated more than 4-fold; those that are within yellow boxes are downregulated more than 2-fold but less than 4-fold; those not within boxes are not significantly differentially expressed and those within blue boxes are upregulated more than 2-fold. None of these genes were upregulated more than 4-fold. All values are arithmetic.

The Stu1 transcription factor was downregulated 5-fold in spherules. This transcription factor was found to be required for optimal hyphal growth in *H. capsulatum* [30], so the difference between expression of this gene in the mycelial and parasitic form is shared by these two pathogenic, dimorphic fungi. However, direct experiments about the role of Stu1 have not been done in *Coccidioides* spp. Eleven out of 29 C2H2 type zinc finger domain-encoding proteins were also downregulated more than 2-fold and only four were upregulated. The downregulated genes are clustered together within a phylogenetic tree of the *C. immitis* C2H2 transcription factors suggesting that they may share functions (Figure 3).

The expression of HLH transcription factors was also downregulated. Both C2H2 and HLH transcription factors influence growth rate and differentiation in *Neurospora crassa* [31]. Most transcription factors containing the fungal Zn(2)–Cys(6) binuclear cluster domain, the most common class of zinc finger protein in the *C*. *immitis*., are not differentially expressed. In *H. capsulatum,* four transcription factors (Ryp1, Ryp2, Ryp3 and Ryp4) are required for differentiation into yeast [32]. These transcription factors are needed for changes in the transcriptional responses to an increase in temperature that triggers yeast formation [28]. The *C. immitis* homologs of these proteins, Ryp2 and Ryp4 (also known as FacB), are upregulated 2.7- and 4.72-fold in spherules, but the other transcription factor is not. These factors are critical regulators of yeast morphology and fungal virulence in *H. capsulatum* [33]. The upregulation of Ryp2 and Ryp4 in *C. immitis* suggests that this regulator may play a similar role in the *Coccidioides* spp. as it does in *H. capsulatum*.

Thirty-four pathways were enriched in downregulated genes (*p* < 0.01); five had a Benjamini–Hochberg *p* adjusted value < 0.05. Several pathways involved in detoxification of compounds by glutathione are highly enriched. Other enriched pathways included metabolism of xenobiotics by cytochrome p450, metabolism of chloroalkane and methane. This suggests that the mycelial form may encounter more toxic compounds than spherules. Another interesting observation is that the ergosterol pathway is enriched in downregulated genes, which is consistent with the GO term enrichment results and suggests that mycelia and spherules may have somewhat different susceptibilities to azole antifungal drugs.

### 3.2. Comparison to of Differential Gene Expression in Young Spherules to Previous Studies in Coccidioides Immitis

The RNA used in this study was derived from mycelia and spherules obtained from frozen samples of spherules and mycelia obtained from the previous microarray analysis [10]. The microarray study found that 2.5% of the total genes were upregulated more than 4-fold in young spherules; the number of differentially expressed genes in the current RNA-Seq analysis was significantly larger (8%). However, 69% of the differentially expressed genes identified in the microarray study were also differentially expressed in this RNA-Seq study. These results suggest that the RNA-Seq is a more sensitive technique for determining differential expression, but differences observed in microarray tend to be found in RNA-Seq too.

GO enrichment analysis of the up- and downregulated genes were similar in the two studies. Upregulated genes in the microarray study were enriched for oxidation-reduction processes and terms for sulfate and sulfite biosynthesis. Enrichment of metal transporter and homeostasis genes was not recognized in the microarray study. However, GO analysis of the down-regulated genes in the microarray study revealed enrichment of transcription factors. One finding of the microarray study was that 25 protein kinase genes were downregulated in spherules. In the current RNA-Seq study, 16 of these genes were also found to be downregulated.

**Figure 4:**
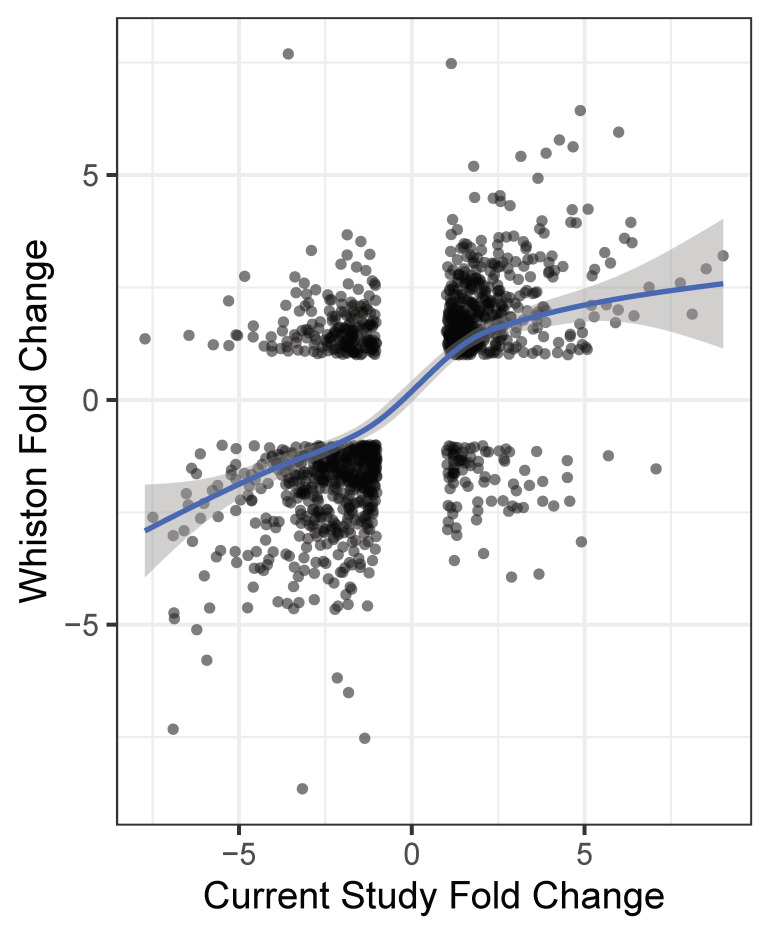
Comparison of FC values in this study to a previous study by Whiston et al. Legend: Genes with log_2_ FC values (young spherules/mycelia) > 2 or < –2 in the current study were selected. FC values of genes in the current study are shown on the X axis and the FC values of matched genes from Whiston study [7] are shown on the Y axis. The regression line was derived from the linear model and the shaded area indicates the 95% confidence limits. The plot was made with ggplot2 R package and the regression line was drawn with the geom smooth option.

Whiston has previously published a study comparing *C. immitis* spherules (Day 4 maturity) to mycelia [7]. Reanalyzing Whiston’s data using our Kallisto/DESeq2 pipeline, we found that 902 genes were differentially expressed by at least 2 spherule/mycelia FC in both studies. The FC values for differentially expressed genes in the Whiston study and young spherules/mycelia in this study are compared in Figure 4. There is a positive correlation between the two studies (73% of the genes are in the same quadrants) but there are also obvious exceptions. The difference in spherule maturity may account for some of these disparities.

### 3.3. Differential Gene Expression in Mature Spherules vs. Mycelia

**Figure 5:**
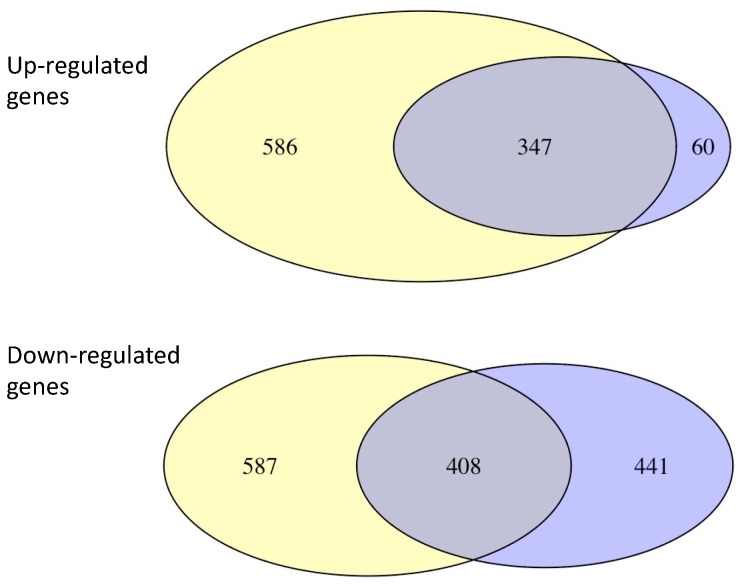
Overlap of up- and downregulated genes in young and mature spherules. Legend: Comparison of up- and downregulated genes in young spherules and mature spherules. Blue represents young spherules, yellow represents mature spherules.

Gene expression in mature spherules was also compared to the gene expression in mycelia (Supplemental Table S1). There were many more genes upregulated in mature spherules (960 genes) than in young ones (408 genes), but the great majority of the genes upregulated in young spherules were also upregulated in mature organisms. In contrast, there were many genes that were downregulated in young spherules but not mature spherules and vice versa (Figure 5). Genes that were differentially expressed in both young and mature spherules had very similar FC values (Supplemental Figure S1). This suggests that upregulation of some genes may be needed for all maturities of the spherule phase.

The enrichment of GO terms in the upregulated genes were similar to the results in young spherules; oxidation-reduction, transmembrane transport and integral membrane component terms were highly enriched (Supplemental Table S2). In contrast, one of the most significantly enriched GO terms in the genes that were downregulated in mature spherules were associated with microtube activity and kinesin. The functional consequences of downregulation of microtubule genes are very difficult to predict, since they play so many different roles in cell biology. The cellular component GO term for the cell wall was also highly enriched in the downregulated genes.

### 3.4. Mature Spherules Compared to Young Spherules

Gene expression in mature spherules was also compared to that in young spherules. A relatively small number of genes were differentially expressed. The most differentially expressed genes are in Table 4.

The most highly upregulated gene is a homolog of Hsp31, which is a methylglyoxalase, a chaperone stress-response gene in yeast [34]. The expression of this gene increases in response to DNA replication stress [34]. A ferritin-like protein, involved in iron regulation and oxidation reactions is also upregulated, as are genes involved in mRNA splicing, transcription, and meiotic recombination. Expression of alpha-amylase-1 gene was also up-regulated; this gene is required for pathogenicity in *H. capsulatum*, but its role in the pathogenicity of *C. immitis* is unknown [35]. The most dramatically downregulated gene in mature spherules was tyrosinase, a gene that plays a central role in the synthesis of melanin. The beta-glucan synthesis-associated protein Kre6 and beta-glucosidase, two genes that influence beta-glucan metabolism were also downregulated, as were two other genes influencing DNA replication (cell division cycle protein Cdc20 and DNA replication helicase Dna2). The downregulation of these genes suggests that remodeling of the cell wall and DNA synthesis decrease in mature spherules.

### 3.5. Expression of Transposable Elements in Young Spherules Compared to Mycelia

We have previously identified 1309 *Gypsy*, *Copia* and *TcMar* transposons in the *C. immitis* RS genome and found that proximity to a transposable element (TE) was associated with a lower level of protein-encoding gene expression in *C. immitis* mycelia [31]. In this study, we compared expression of TE mRNA in mycelia and young spherules. Only 350 TE genes had an adjusted *p* < 0.05, and the median baseMean of these genes was 20.4, indicating most TEs were poorly expressed. A total of 230 were upregulated in young spherules and only 15 were downregulated. Gypsy TE were the most common upregulated transposons. Analysis of upregulated predicted protein-encoding genes in young spherules showed that a few of them are transposon proteins. A previous proteomic study found one transposon protein in *C. posadasii* spherules [32]. There was a total of 77 genes that were within 1 kb up- or downstream of the upregulated TE. The median FC of these genes was 3.90, so genes near the up-regulated TE were somewhat overexpressed in spherules.

## 4. Summary

The transition from the environmental arthroconidia/mycelial form to the spherule is required for the pathogenesis of *Coccidioides* spp. The morphologic changes are dramatic—the organism changes from a mycelium with internal spores to a round structure that enlarges circumferentially and divides internally to form a large number of endospores [36]; these are released and can differentiate into mature spherules. The importance of spherule maturation and spherule release is demonstrated by the observation that an engineered chitinase deletion mutant that does not form spherules is avirulent [37]. For these reasons, understanding the transcription program of spherules as they mature is clearly important for comprehending the biology of this dimorphic fungus.

Our findings in this study confirm previous experiments in showing that there are substantial differences in gene expression in spherules and mycelia [7,10]. One of the most interesting findings of this study is that differentially expressed genes are more likely to be genus-specific than those that are not. Although the function of these genes in unknown, and they likely have evolved fairly recently, the results in our study suggest that they play an important role in mycelial/spherule differentiation.

The expression of copper and iron transporters is upregulated in spherules, which is consistent with previous studies in *C. posadasii* [21] and *H. capsulatum* [23]. Two of the four Ryp transcription factor genes that have been found to be important for yeast formation and virulence in *H. capsulatum* are also upregulated in *C. immitis*, suggesting that these two organisms may use similar transcription programs for phase differentiation.

Another interesting group of genes that is downregulated in young spherules are the transcription factors. The APSES family transcription factor Stu1, several HLH and many C2H2 transcription factors are downregulated. Identification of the genetic programs driven by these transcription factors in *C. immitis* should be useful for understanding the biological significance of these changes.

Analysis of mature spherules shows that almost all the upregulated genes in young spherules remain upregulated. This suggests that this group of genes may be required for life in the spherule phase; examining the transcriptome of spherules of intermediate maturities would address this question. Furthermore, there were a very small number of differentially expressed genes between the two spherule maturities, which also suggests that young and mature spherules have very similar transcription programs. This study confirms that many genes are differentially expressed in mycelia and spherules and suggests that further investigation of the expression profiles of these morphologic forms may be useful for understanding the biology of this organism.

## Figures and Tables

**Table 1:** Most differentially expressed genes in young spherules compared to mycelia.

Gene ID	Product Description	Ortholog Count	Length	# TM Domains	SignalP	FC (log_2_ Values)	Genus-Specific
Upregulated							
CIMG_04613	Hypothetical protein—SOWgp	3	324	0	Y	9.012	Y
CIMG_10264	Aldehyde reductase	1176	314	0	N	8.116	N
CIMG_09753	ABC multidrug transporter	569	1501	16	N	7.773	N
CIMG_10032	Expression library immunization antigen 1-surface protein antigen	96	224	0	Y	7.092	N
CIMG_10037	Copper transporter	121	193	2	N	7.072	N
CIMG_01115	Thiamine thiazole synthase, thiamine thiazole synthase, variant	170	328	0	N	6.901	N
CIMG_08103	Opsin 1	214	289	7	N	6.870	N
CIMG_06250	Methyltransferase domain-containing protein	51	320	1	Y	6.431	Y
CIMG_11522	Hypothetical protein—genus-specific	2	85	1	N	6.376	Y
CIMG_10007	Hypothetical protein—genus-specific	3	125	0	N	6.341	Y
CIMG_02628	Arp2/3 complex subunit Arc16	199	312	0	N	6.158	N
CIMG_09539	Hypothetical	136	387	0	N	5.989	N
CIMG_12484	hypothetical protein—genus-specific	1	124	0	N	5.977	Y
CIMG_09750	Amino acid adenylation domain-containing protein	1262	8032	0	N	5.898	N
CIMG_05758	parasitic phase-specific protein PSP-1	458	279	7	N	5.752	N
CIMG_11264	Hypothetical protein	59	76	1	Y	5.693	N
CIMG_00534	Glycerate kinase	37	437	1	N	5.635	N
CIMG_10013	Hypothetical protein—genus-specific	0	114	0	N	5.586	Y
CIMG_01211	Putative major facilitator superfamily (MFS) transporter	11	553	13	N	5.294	N
Downregulated							
CIMG_03418	CBF1-interacting co-repressor CIR domain-containing protein	99	336	0	N	−8.094	N
CIMG_01310	4-hydroxyphenylpyruvate dioxygenase	183	399	0	N	−7.717	N
CIMG_07209	Life-span regulatory factor domain-containing protein	56	151	0	N	−7.491	N
CIMG_03873	Protoglobin domain-containing protein	136	245	0	N	−7.320	N
CIMG_07928	SAM and PH domain-containing protein	74	795	0	N	−6.970	N
CIMG_03716	Heat shock protein 30	115	238	0	N	−6.904	N
CIMG_00925	Hypothetical protein—cell wall protein	82	157	0	Y	−6.903	N
CIMG_00284	Hypothetical protein	5	118	0	Y	−6.898	Y
CIMG_09232	Hypothetical protein	59	571	1	Y	−6.880	Y
CIMG_00099	C2H2 finger domain-containing protein	116	595	0	N	−6.868	N
CIMG_08613	Metalloproteinase 7	4	357	0	Y	−6.746	N
CIMG_12190	Hypothetical protein—genus-specific	1	137	0	N	−6.689	Y
CIMG_03848	DnaJ domain-containing protein	68	536	0	N	−6.660	N
CIMG_13167	Beta-glucosidase 5	94	489	0	N	−6.588	N
CIMG_13284	Hypothetical protein—genus-specific	0	74	0	N	−6.523	Y
CIMG_04597	GPI anchored serine-threonine rich protein	68	216	0	Y	−6.467	Y
CIMG_00348	Endochitinase 2	317	897	0	Y	−6.445	N
CIMG_10468	Hypothetical protein—genus-specific	3	132	0	N	−6.370	Y
CIMG_00302	Hypothetical protein—genus-specific	74	228	0	Y	−6.345	Y
CIMG_07925	Hypothetical protein—genus-specific	3	168	0	N	−6.229	Y

Legend Table 1: The 20 most up- and downregulated genes in young spherules. The gene ID, product description, ortholog count, length, number of transmembrane domains (TM), presence of a signal peptide Yes (Y) or No (N), fold change log_2_ expression ratios of young spherules/young spherules and whether the gene is genus-specific Yes (y) No (Y) is shown SOWgp: spherule outer wall glycoprotein.

**Table 2:** Genus-specific genes.

Group	Total	Genus-Specific	%
All genes	9759	2305	24
Upregulated	407	127	31^a^
Downregulated	849	329	39 ^a^

Legend Table 2: Comparison of the number of differentially expressed (young spherule/mycelia) genus-specific genes to all genus-specific genes. a) *p* value < 0.05 (Chi-square test) comparing the fraction of genus-specific genes in up- or downregulated genes to the fraction in all genes.

**Table 3:** Relative expression of transcription factors in young spherules and mycelia.

Transcription Factor	Total Number	Number (Adjusted *p* < 0.05)	PFAM	FC (log_2_ Young Spherule/Mycelium)			
				>2	>1	<−1	<−2
APSES	5	3	PF13637			2	1
HLH transcription factor	8	3	PF00010			2	1
C2H2 type zinc finger domain-containing protein	29	21	PF00096		1	11	7
fungal Zn(2)–Cys(6) binuclear cluster domain-containing	91	51	PF00172	4	16	17	7
Homeobox-like domain-containing protein	6	6	PF00249	2			
Other Regulatory Proteins							
ryp family	4	3	PF09729; PF11754 PF00172; PF04082	1	2	1	

Legend: Differential expression of transcription factors in spherules compared to mycelia. All FC values are expressed as log2.

**Table 4:** Most differentially expressed genes in mature spherules compared to young spherules.

Gene ID	Product Description	Ortholog Count	Length	# TM Domains	SignalP	FC (log_2_ Values)
Upregulated						
CIMG_03805	Possible HSP31	281	243	0	N	4.626
CIMG_12285	hypothetical protein	10	151	0	N	4.074
CIMG_08736	hypothetical protein	5	156	0	N	3.705
CIMG_07646	hypothetical protein	3	350	0	N	3.606
CIMG_07274	Ferritin-like domain-containing protein	105	455	0	Y	3.507
CIMG_13581	hypothetical protein	0	71	0	N	3.306
CIMG_05456	Possible pre-mRNA-splicing factor cef1	45	237	0	N	3.281
CIMG_08524	Dynamin family protein	450	711	0	N	3.186
CIMG_02168	Possible transcription factor wer	12	444	0	N	3.140
CIMG_00573	Cytochrome P450 51	236	511	2	N	3.064
CIMG_08156	Possible meiotic recombination protein dmc1/lim15 homolog	91	463	0	N	3.058
CIMG_04521	DUF1399 domain-containing protein	195	745	0	N	2.984
CIMG_03465	hypothetical protein	8	383	0	N	2.907
CIMG_06696	Possible dual oxidase	508	295	2	Y	2.902
CIMG_06262	Possible rest corepressor 1	37	185	0	N	2.900
CIMG_08666	FK506-binding protein 1	202	120	0	N	2.829
CIMG_13473	YjgH family protein	45	206	0	N	2.778
CIMG_04544	Alpha-amylase AmyA	65	582	0	Y	2.769
CIMG_01977	Hypothetical protein	5	482	0	Y	2.740
CIMG_07685	Hypothetical protein	8	105	0	N	2.711
Downregulated						
CIMG_07583	Tyrosinase	41	658	0	Y	−4.248
CIMG_03173	Hypothetical protein	5	189	1	N	−3.863
CIMG_00927	Acyl–CoA synthetase	137	693	1	N	−3.204
CIMG_05421	Beta-glucan synthesis-associated protein KRE6	222	642	1	N	−3.046
CIMG_03257	Cell division cycle protein Cdc20	172	599	0	N	−3.030
CIMG_03517	Hypothetical protein	85	2137	0	N	−2.887
CIMG_01729	Aconitate hydratase, mitochondrial	352	784	0	N	−2.844
CIMG_13686	Hypothetical protein	0	94	0	N	−2.806
CIMG_00254	GTP-binding protein rhoC	109	278	0	N	−2.783
CIMG_03888	Betaglucosidase	1153	858	0	Y	−2.731
CIMG_01317	Dethiobiotin synthase	136	807	0	N	−2.731
CIMG_01369	Myosin rod fragments superfamily protein, putative	1387	1271	0	N	−2.703
CIMG_08907	Cytochrome b2	480	504	0	N	−2.660
CIMG_12964	Hypothetical protein	0	168	0	Y	−2.615
CIMG_00847	GTP binding protein	109	1469	0	N	−2.609
CIMG_05523	Ca2+ regulator and membrane fusion protein Figure 1, putative	111	268	4	Y	−2.552
CIMG_13558	DNA replication helicase Dna2	158	1659	0	N	−2.523
CIMG_08747	Putative tetratricopeptide repeat (TPR)-like superfamily protein	232	702	0	N	−2.493
CIMG_01268	MFS phospholipid transporter	317	496	10	N	−2.492
CIMG_05728	L-serine dehydratase	173	426	0	N	−2.475
CIMG_09628	Protein–tyrosine phosphatase	171	600	0	N	−2.471

Legend: The 20 most up- and downregulated genes in mature spherule compared to young spherules. The gene ID, product description, ortholog count, length, number of transmembrane domains (TM), presence of a signal peptide Yes (Y) or No (N) and fold change log_2_ expression ratios of mature spherules/young spherules are shown.

## Data Availability

The data for this manuscript are available at NCBI GEO, accession number GSE171286.

## Supplementary Materials

The following are available online at https://www.mdpi.com/article/10.3390/jof7050366/s1, Figure S1: Heat map comparing a sample of FC values of young spherule/mycelia to mature spherules/mycelia, Supplemental Table S1: Relative expression (FC) of young spherules/mycelia, Supplemental Table S2: GO enrichment analysis of differentially expressed young spherule genes/mycelia and mature spherules/mycelia, Supplemental Table S3: Pathway enrichment of differentially expressed young spherules/mycelia, Supplemental Table S4: Relative expression (FC) of mature spherule/mycelia, Supplemental Table S5: Relative expression (FC) of mature spherule/young spherules, Supplemental Table S6: Relative expression (FC) of TE in young spherules/mycelia.

## References

[1] Engelthaler D.M., Roe C.C., Hepp C.M., Teixeira M., Driebe E.M., Schupp J.M., Gade L., Waddell V., Komatsu K., Arathoon E. (2016). Local Population Structure and Patterns of Western Hemisphere Dispersal for *Coccidioides* spp., the Fungal Cause of Valley Fever. MBio.

[2] Kirkland T.N., Fierer J. (2018). *Coccidioides immitis* and *posadasii*; A review of their biology, genomics, pathogenesis, and host immunity. Virulence.

[3] Nguyen C., Barker B.M., Hoover S., Nix D.E., Ampel N.M., Frelinger J.A., Orbach M.J., Galgiani J.N. (2013). Recent advances in our understanding of the environmental, epidemiological, immunological, and clinical dimensions of coccidioidomycosis. Clin. Microbiol. Rev..

[4] Whiston E., Taylor J.W. (2014). Genomics in *Coccidioides*: Insights into evolution, ecology, and pathogenesis. Med. Mycol..

[5] Converse J.L. (1956). Effect of physico-chemical environment of spherulation of *Coccidioides immitis* in a chemically defined medium. J. Bacteriol..

[6] Mead H.L., Van Dyke M.C.C., Barker B.M. (2020). Proper Care and Feeding of Coccidioides: A Laboratorian’s Guide to Cultivating the Dimorphic Stages of *C. immitis* and *C. posadasii*. Curr. Protoc. Microbiol..

[7] Whiston E., Zhang Wise H., Sharpton T.J., Jui G., Cole G.T., Taylor J.W. (2012). Comparative transcriptomics of the saprobic and parasitic growth phases in *Coccidioides* spp. PLoS ONE.

[8] Cole G.T., Kirkland T., Franco M., Zhu S.W., Yuan L., Sun S.H., Hearn V.N. (1988). Immunoreactivity of a surface wall fraction produced by spherules of *Coccidioides immitis*. Infect. Immun..

[9] Hung C.Y., Yu J., Seshan K.R., Reichard U., Cole G.T. (2002). A parasitic phase-specific adhesin of *Coccidioides immitis* contributes to the virulence of this respiratory fungal pathogen. Infect. Immun..

[10] Viriyakosol S., Singhania A., Fierer J., Goldberg J., Kirkland T.N., Woelk C.H. (2013). Gene expression in human fungal pathogen *Coccidioides immitis* changes as arthroconidia differentiate into spherules and mature. BMC Microbiol..

[11] Bray N.L., Pimentel H., Melsted P., Pachter L. (2016). Near-optimal probabilistic RNA-seq quantification. Nat. Biotechnol..

[12] Basenko E.Y., Pulman J.A., Shanmugasundram A., Harb O.S., Crouch K., Starns D., Warrenfeltz S., Aurrecoechea C., Stoeckert C.J., Kissinger J.C. (2018). FungiDB: An Integrated Bioinformatic Resource for Fungi and Oomycetes. J. Fungi.

[13] Li L., Stoeckert C.J., Roos D.S. (2003). OrthoMCL: Identification of ortholog groups for eukaryotic genomes. Genome Res..

[14] Sievers F., Wilm A., Dineen D., Gibson T.J., Karplus K., Li W., Lopez R., McWilliam H., Remmert M., Soding J. (2011). Fast, scalable generation of high-quality protein multiple sequence alignments using Clustal Omega. Mol. Syst. Biol..

[15] Priebe S., Kreisel C., Horn F., Guthke R., Linde J. (2015). FungiFun2: A comprehensive online resource for systematic analysis of gene lists from fungal species. Bioinformatics.

[16] Karp P.D., Billington R., Caspi R., Fulcher C.A., Latendresse M., Kothari A., Keseler I.M., Krummenacker M., Midford P.E., Ong Q. (2019). The BioCyc collection of microbial genomes and metabolic pathways. Brief Bioinform..

[17] Kanehisa M., Furumichi M., Tanabe M., Sato Y., Morishima K. (2017). KEGG: New perspectives on genomes, pathways, diseases and drugs. Nucleic Acids Res..

[18] Ivey F.D., Magee D.M., Woitaske M.D., Johnston S.A., Cox R.A. (2003). Identification of a protective antigen of *Coccidioides immitis* by expression library immunization. Vaccine.

[19] Delgado N., Hung C.Y., Tarcha E., Gardner M.J., Cole G.T. (2004). Profiling gene expression in *Coccidioides posadasii*. Med. Mycol..

[20] Petito G., de Curcio J.S., Pereira M., Bailão A.M., Paccez J.D., Tristão G.B., de Morais C.O.B., de Souza M.V., de Castro Moreira Santos A., Fontes W. (2020). Metabolic Adaptation of *Paracoccidioides brasiliensis* in Response to in vitro Copper Deprivation. Front. Microbiol..

[21] Mead H.L., Roe C.C., Higgins Keppler E.A., Van Dyke M.C.C., Laux K.L., Funke A.L., Miller K.J., Bean H.D., Sahl J.W., Barker B.M. (2020). Defining Critical Genes During Spherule Remodeling and Endospore Development in the Fungal Pathogen, *Coccidioides posadasii*. Front. Genet..

[22] Youseff B.H., Holbrook E.D., Smolnycki K.A., Rappleye C.A. (2012). Extracellular superoxide dismutase protects *Histoplasma* yeast cells from host-derived oxidative stress. PLoS Pathog..

[23] Hwang L.H., Seth E., Gilmore S.A., Sil A. (2012). SRE1 regulates iron-dependent and -independent pathways in the fungal pathogen *Histoplasma capsulatum*. Eukaryot. Cell.

[24] Gauthier G.M., Sullivan T.D., Gallardo S.S., Brandhorst T.T., Vanden Wymelenberg A.J., Cuomo C.A., Suen G., Currie C.R., Klein B.S. (2010). SREB, a GATA transcription factor that directs disparate fates in *Blastomyces dermatitidis* including morphogenesis and siderophore biosynthesis. PLoS Pathog..

[25] Mirbod-Donovan F., Schaller R., Hung C.Y., Xue J., Reichard U., Cole G.T. (2006). Urease produced by *Coccidioides posadasii* contributes to the virulence of this respiratory pathogen. Infect. Immun..

[26] Wise H.Z., Hung C.-Y., Whiston E., Taylor J.W., Cole G.T. (2013). Extracellular ammonia at sites of pulmonary infection with *Coccidioides posadasii* contributes to severity of the respiratory disease. Microb. Pathog..

[27] Kirkland T.N. (2016). A few shared up-regulated genes may influence conidia to yeast transformation in dimorphic fungal pathogens. Med. Mycol..

[28] Boyce K.J., McLauchlan A., Schreider L., Andrianopoulos A. (2015). Intracellular growth is dependent on tyrosine catabolism in the dimorphic fungal pathogen *Penicillium marneffei*. PLoS Pathog..

[29] Felipe M.S.S., Andrade R.V., Arraes F.B.M., Nicola A.M., Maranhão A.Q., Torres F.A.G., Silva-Pereira I., Poças-Fonseca M.J., Campos E.G., Moraes L.M.P. (2005). Transcriptional profiles of the human pathogenic fungus *Paracoccidioides brasiliensis* in mycelium and yeast cells. J. Biol. Chem..

[30] Longo L.V.G., Ray S.C., Puccia R., Rappleye C.A. (2018). Characterization of the APSES-family transcriptional regulators of *Histoplasma capsulatum*. FEMS Yeast Res..

[31] Carrillo A.J., Schacht P., Cabrera I.E., Blahut J., Prudhomme L., Dietrich S., Bekman T., Mei J., Carrera C., Chen V. (2017). Functional Profiling of Transcription Factor Genes in *Neurospora crassa*. G3.

[32] Webster R.H., Sil A. (2008). Conserved factors Ryp2 and Ryp3 control cell morphology and infectious spore formation in the fungal pathogen *Histoplasma capsulatum*. Proc. Natl. Acad. Sci. USA.

[33] Rodriguez L., Voorhies M., Gilmore S., Beyhan S., Myint A., Sil A. (2019). Opposing signaling pathways regulate morphology in response to temperature in the fungal pathogen *Histoplasma capsulatum*. PLoS Biol..

[34] Bankapalli K., Saladi S., Awadia S.S., Goswami A.V., Samaddar M., D’Silva P. (2015). Robust glyoxalase activity of Hsp31, a ThiJ/DJ-1/PfpI family member protein, is critical for oxidative stress resistance in *Saccharomyces cerevisiae*. J. Biol. Chem..

[35] de Vries R.P., Riley R., Wiebenga A., Aguilar-Osorio G., Amillis S., Uchima C.A., Anderluh G., Asadollahi M., Askin M., Barry K. (2017). Comparative genomics reveals high biological diversity and specific adaptations in the industrially and medically important fungal genus *Aspergillus*. Genome Biol..

[36] Cole G.T., Pope L.M., Huppert M., Sun S.H., Starr P. (1983). Ultrastructure and composition of conidial wall fractions of *Coccidioides immitis*. Exp. Mycol..

[37] Xue J., Chen X., Selby D., Hung C.Y., Yu J.J., Cole G.T. (2009). A genetically engineered live attenuated vaccine of *Coccidioides posadasii* protects BALB/c mice against coccidioidomycosis. Infect. Immun..

