# Supplementary figures and images for: Transcriptional Analysis of Coccidioides immitis Mycelia and Spherules by RNA Sequencing
Source: J Fungi (Basel). 2021 May 7;7(5):366. doi: 10.3390/jof7050366 (PMC8150946; doi:10.3390/jof7050366)

# Spherule/Mycelia Fold Change (log 2)

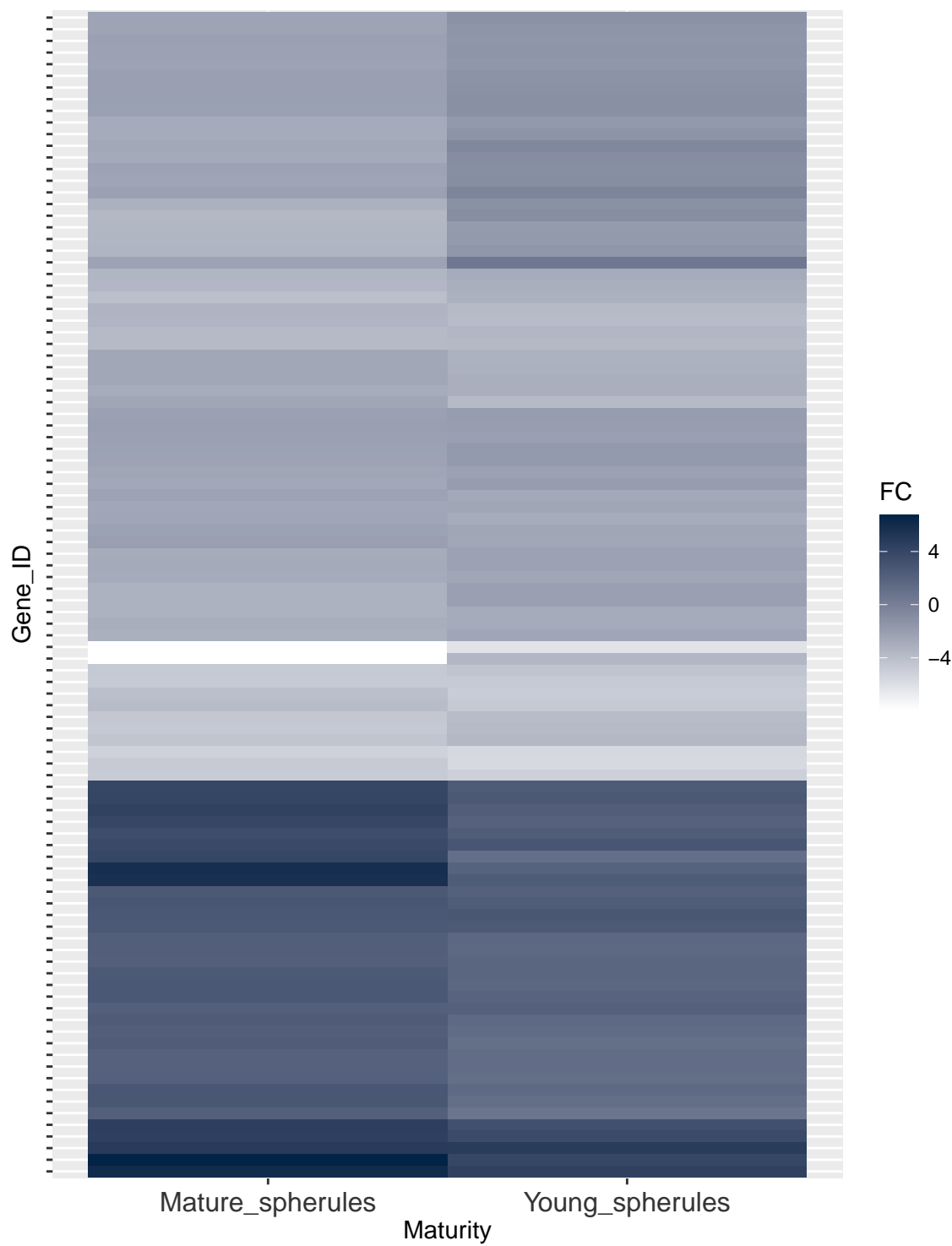

Supplement: Supplementary file 1 [file jof-07-00366-s001.zip › jof-1180561-Supplemental Figure S1.pdf]
